# BeWo‐Derived Extracellular Vesicles Downregulate IL‐6R*α* Expression via miRNAs on CD4+ T Lymphocytes

**DOI:** 10.1111/aji.70335

**Published:** 2026-10-18

**Authors:** Gábor Seregélyes, Bence Nagy, Árpád Ferenc Kovács, Nóra Fekete, Edit I. Buzás, Éva Pállinger

**Affiliations:** ^1^ Institute of Genetics, Cell‐ and Immunobiology Semmelweis University Budapest Hungary; ^2^ Heart and Vascular Center Semmelweis University Budapest Hungary; ^3^ Department of Pathology and Experimental Cancer Research Semmelweis University Budapest Hungary; ^4^ MTA‐SE Immune‐Proteogenomics Extracellular Vesicle Research Group Budapest Hungary; ^5^ HCEMM‐SE Extracellular Vesicle Research Group Budapest Hungary

**Keywords:** CD4+ T cells, extracellular vesicles, human pregnancy, immune tolerance, interleukin‐6, IL‐6R*α*, IL‐6 pathway, miRNA, regulatory T cells

## Abstract

**Problem:**

A balanced Th1/Th2/Th17/Treg status is necessary for a successful pregnancy. T cell polarization depends on the local cytokine environment and the responsiveness of T lymphocytes to cytokines. Trophoblast‐derived extracellular vesicles (EVs) influence the function of maternal T lymphocytes, contributing to their polarization into the Treg phenotype. We examined whether BeWo‐derived EVs affect T cell IL‐6 responsiveness using the BeWo cell line as an experimental model and characterized the molecular cargo of BeWo ‐EVs that may contribute to this process.

**Methods:**

Circulating lymphocytes from non‐pregnant women were treated with BeWo‐EVs or co‐cultured with BeWo cells. We detected IL‐6R*α* expression by flow cytometry and IL6R signaling pathways by RT‐PCR. The microRNA (miRNA) content of BeWo EVs was identified by sequencing. Finally, we compared IL‐6R*α* expression levels in circulating T lymphocytes from healthy pregnant and non‐pregnant women.

**Results:**

BeWo‐EVs reduced IL‐6R*α* expression on CD4+ T cells. This modifies the IL‐6 pathway by downregulating the transcription factors STAT3, NFKB1, and PIAS3 while upregulating STAT1. This may be due to the presence of specific microRNAs (miRNAs), such as hsa‐mir‐92a‐3p, hsa‐mir‐520f‐3p, and hsa‐mir‐25‐3p, in BeWo‐EVs that target the IL‐6 pathway. Treatment with BeWo‐EVs also induced phenotypic and functional changes in T cells and enhanced the ratio of CD4+/CD25+ T cells that produce IL‐10. Pregnancy‐associated IL‐6R*α* downregulation has been demonstrated in clinical samples. Significantly lower levels of IL‐6R*α* were detected on circulating CD4+CD25+ T cells in healthy pregnant women compared to healthy non‐pregnant individuals.

**Conclusions:**

Our studies suggest that the microRNA content of trophoblast‐derived EVs significantly influences the local polarization of maternal T lymphocytes by modifying the IL‐6R*α* signaling pathway.

**Abbreviations**
12.5K lEV: intermediate‐sized extracellular vesicles
EV: extracellular vesicles
FSC: Forward Scatter
HLA‐G: human leukocyte antigen G (major histocompatibility complex, class I, G)
IL‐6R*α*
: interleukin 6 receptor alpha
miRNA: micro ribonucleic acid
PS: phosphatidylserine
PSR: phosphatidylserine receptor
sEV: small‐sized extracellular vesicles
SSC: Side Scatter

## 1 Introduction

The adaptation of the maternal immune system to pregnancy is strictly regulated and can be characterized by immunological stages, or the “immune clock” [1]. Recognizing the semi‐allogeneic fetus and developing maternal immune tolerance are prerequisites for a successful pregnancy. Although an inflammatory environment is necessary for embryo implantation, the immune system transitions to an anti‐inflammatory state by the end of the first trimester [2]. The presence of regulatory T cells (Treg) and Th2 cells in the placenta is essential for fetal growth [3, 4, 5, 6]. Alterations to this immune milieu can result in recurrent spontaneous abortion [7, 8]. The local cytokine environment determines the polarization of naïve CD4+ T helper (Th) cells into specific subsets, such as Th1, Th2, Th17, and Treg cells. Transforming growth factor beta (TGF‐*β*) and interleukin 2 (IL‐2) are critical for Treg differentiation. The final effects of TGF‐*β* signaling are also highly influenced by surrounding cytokines. Proinflammatory cytokines, such as IL‐1*β*, IL‐6, and TNF‐*α*, can neutralize the effects of TGF‐*β* and favor Th17 development over Treg polarization [9, 10]. IL‐6 is a Janus‐faced player during pregnancy [11]. Although IL‐6 is present in the amniotic fluid and the placenta, already from the earliest period of pregnancy [12, 13], the complete role of it has not been clarified yet. According to our knowledge IL‐6, as a pleiotropic cytokine contributes to the main physiological processes during pregnancy, including implantation, embryogenesis and parturition [14]. On the other hand, IL‐6 directly acts on CD4+ Th cells and plays a role in the orchestration of Th cell differentiation and function [15, 16].

The tolerogenic immune environment at the feto‐maternal interface is maintained through direct cell‐to‐cell interactions, soluble mediators, and extracellular vesicles (EVs) [17, 18]. The presence of human leukocyte antigen‐G (HLA‐G) ‐positive trophoblast‐derived EVs at the feto‐maternal interface and in the maternal bloodstream is a classic feature of pregnancy [19, 20, 21]. EVs are membrane‐covered intercellular messengers that are constitutively secreted by eukaryotic and prokaryotic cells [22]. Two types of EVs can be distinguished according to their production: ectosomes and exosomes. However, available isolation methods separate them based on size [23]. In this context, the following definitions apply to EV populations: small EVs (sEVs), which have an average diameter under 200 nm and are enriched in exosomes, and large EVs, which have an average diameter higher than 200 nm. The large EV fraction contains the “12.5K lEVs” (formerly known as iEVs or microvesicles), which have been isolated and purified by centrifugation at 12,500 *g* [24]. These EV subpopulations may differ in their membrane components, including proteins and lipids, as well as in their cargo, such as nucleic acids (e.g., microRNAs, intact mRNAs, long non‐coding RNAs, and double‐stranded DNA) [25]. Placental EVs are critical modulators of the maternal immune system [26]. Because EVs are involved in many physiological and pathological processes, they can serve as diagnostic and prognostic biomarkers for various pathological conditions [27]. In our previous study, we found that both platelet‐ and trophoblast‐derived EVs target immune cells [28] and trophoblastic‐derived 12.5K lEVs induced Treg expansion is associated with the HSPE1 expression and the specific miRNA cargo of EVs [29].

In the present study, we demonstrate that trophoblast‐derived EVs modulate the IL‐6 sensitivity of Th cells by regulating their interleukin‐6 receptor alpha (IL‐6R*α*) expression via miRNA, thereby playing an important role in local Treg cell polarization.

## 2 Material and Methods

### 2.1 Patients and Sample Collection

Peripheral venous blood samples were collected from the median cubital vein of 37 healthy pregnant women in their 18th week of pregnancy and 15 healthy non‐pregnant women using Vacutainer Brand Plus Sodium Heparin Tubes or Vacutainer EDTA Tubes from Becton Dickinson (BD, San Jose, California, USA). The blood samples arrived at the laboratory within two hours and were immediately processed. The pregnant women underwent routine obstetrical control at the Department of Obstetrics and Gynecology at Semmelweis University's Faculty of Medicine. An obstetrical history of previous spontaneous abortions, the course of previous pregnancies, hypertension, gestational diabetes, and preeclampsia were exclusion criteria. Samples from the healthy non‐pregnant women were obtained from the Hungarian National Blood Transfusion Service. The clinical data of the studied groups are presented in Table S1. The study was approved by the Ethics Committee of Scientific Research of Hungary (ETT‐TUKEB: 10147‐4/2015/EKU (93/2014)).

### 2.2 Lymphocyte Separation

Peripheral blood mononuclear cells (PBMCs) were isolated using density gradient centrifugation with Histopaque 1077 (Sigma‐Aldrich, St. Louis, Missouri), and the monocytes were removed by adherence.

### 2.3 Cell Culture (BeWo Cells and Human Lymphocyte Co‐Culturing)

To study the interaction between BeWo cells and lymphocytes, we created a transwell system. We placed 2 × 10^5^ BeWo cells and 1 × 10^6^ lymphocytes in the wells of a BD Falcon 12‐well cell culture insert companion plate (BD, San Jose, CA, USA) in 2 mL of culture media and incubated for 24 h. To exclude the effects of direct cell‐to‐cell interactions, the cells were separated using 1 µm BD Falcon cell culture insert pores. Since both EVs and soluble mediators can pass through the membrane, we treated with isolated BeWo EVs alone to characterize their effects. We used two control groups: one with lymphocytes cultured alone and one with lymphocytes cultured together with BeWo cells (Figure S1).

The BeWo cells were routinely tested for mycoplasma infection. (Figure S2).

### 2.4 Isolation of BeWo‐Derived Large Vesicles (12.5K lEVs) by Differential Centrifugation

2 × 10^6^ BeWo cells were seeded in 75 cm^2^ tissue culture treated flasks in 10 mL of culture media. Once the cells reached 80–90% confluence, we discarded the culture medium and replaced it with 10 mL of FBS‐free medium. Then, we cultured the cells for an additional 24 h. We applied the differential centrifugation method for EV isolation, which yields intermediate recovery and specificity. Next, BeWo cell‐culture supernatant was centrifuged at 300 *g* for 5 min at room temperature (RT) to remove cell debris. The obtained supernatant was centrifuged at 2 000 *g* for 15 min to remove the apoptotic body fraction. The supernatant of the 2 000 *g* speed centrifugation was sedimented at 12 500 *g* for 20 min at 16°C (Z216 MK Microlite centrifuge, fixed angle 200.88 rotor, Hermle Labortechnik GmbH, Wehingen, Germany) and subsequently washed with 0.20 µm filtered phosphate‐buffered saline (PBS) at 12 500 *g* for 15 min at 16°C. The obtained pellets enriched in 12.5K lEV fraction (12.5K pellet) were resuspended in 20–100 µL PBS or serum‐free Ham's F12/K media depending on the downstream analysis (Figure S3).

### 2.5 EV Characterization

Following the recommendations of the International Society for Extracellular Vesicles' (ISEV) latest position paper [24], the isolated EV preparations were characterized by several methods. (Figure S4)

### 2.6 miRNA Sequencing

Three biological replicates of BeWo‐12.5K lEVs were isolated and resuspended in 12 µL of DNase, RNase free water for miRNA sequencing. The RNA integrity was evaluated with Agilent Tapestation and samples with RIN values ≥7 were further processed. For library preparation multiplex Small RNA Libr. Prep Kit was used according to the manufacturer's instructions. The sequencing was performed on an Illumina MiSeq instrument and the paired‐end read value was > 20 million/sample. Next, we aligned the transcriptome sequence reads to the reference genome (Ensembl GRCh38 release) with STAR version 2.5.3a using 2‐pass alignment mode. The mirBase annotation was used in both mapping and read counting. After alignment, the reads were associated with known miRNAs and the number of reads assigned to each miRNA was counted using featureCounts from R package Rsubread. The data were normalized using the TMM normalization method of the edgeR R/Bioconductor package (R version 3.4.1, Bioconductor version 3.5). After pre‐processing and quality control we analyzed the target genes of the miRNAs (miRDB target score ≥80) and the IL‐6R pathway targeting miRNAs with Funrich 3.1.3 [33], g:Profiler [30] and NetworkAnalyst 3.0 [34]. The miRNA‐Seq data has been deposited to the ExoCarta database [35].

### 2.7 Investigation of Target Cells of BeWo‐12.5K lEVs and Characterization of Molecular Interactions between 12.5K lEVs and Lymphocytes

One hundred µL of the separated 12.5K lEVs were labeled with a freshly prepared PKH26 fluorescent dye solution at a final concentration of 5 µM according to the manufacturer's instructions (Merck, USA). After staining, the labeled vesicles were washed by adding 1 mL of RPMI medium and centrifuging at 12,500 *g* for 15 min at 16°C. We evaluated the effectiveness of staining by flow cytometry. Validation was performed using a mock control (the same staining procedure without adding EV samples) and differential detergent lysis (Figure S5). The number of EVs was determined by adding internal counting beads (PKH Reference Beads, Merck, USA). A constant amount of BeWo‐derived PKH labelled 12.5K lEVs (1 × 10^6^) were added to immunophenotyped lymphocytes (5 × 10^5^) for 30 min at 4°C to investigate EV binding. T lymphocyte subsets were defined by CD3+/CD4+ (Th cells), CD3+/CD4+/CD25+ (activated Th and regulatory Th cells) and CD3+/CD8+ (Tc cells) positivity. B cells were characterized by their CD3‐/CD19+ expression, and NK cells by their CD3‐/CD56+ immunophenotype. Antibodies were titrated for the optimal concentration. At the end of the incubation period, cells were washed with PBS and analyzed by flow cytometry.

To characterize the molecular interactions between EVs and lymphocytes, we measured the binding of PKH‐labeled EVs after masking surface PS, CD95L, or HLA‐G molecules using annexin V, anti‐CD95L, or anti‐HLA‐G antibodies. On the other hand, the surface phosphatidylserine receptor (PSR) and CD95 molecules on lymphocytes were also blocked with anti‐PSR polyclonal antibody or anti‐CD95 antibodies. PKH‐labeled BeWo‐derived 12.5K lEVs and immunophenotyped lymphocytes were incubated together in the presence or in absence of monoclonal antibodies in PBS containing 4% bovine serum albumin (BSA) for 30 min at 4°C.

### 2.8 Investigation of IL‐4, IL‐6, IL‐17 and IFNγ Production by *Cytometric Bead Array (CBA)*

IL‐4, IL‐6, IL‐17 and IFN‐γ production was detected from cell culture supernatants, by the BD CBA (BD San Jose, California, USA) system according to the manufacturer's instructions (BD Cytometric Bead Array (CBA) Human Th1/Th2/Th17 Cytokine Kit).

### 2.9 Intracellular IL‐10 Staining of CD4+ T Lymphocytes

5 × 10^5^ human lymphocytes were stimulated with BeWo‐12.5K lEVs for 24 h in the presence of the protein transport inhibitor Brefeldin A (Thermo Fisher Scientific, USA). Stimulated cells were washed and incubated in 100 µL of staining buffer (PBS containing 1% BSA) with a pre‐titrated optimal concentration of PerCP‐conjugated anti‐human CD4 monoclonal antibody (15 min at RT). The immunophenotyped cells were fixed in a 4% paraformaldehyde solution (Sigma‐Aldrich Co., St. Louis, MO) for 10 min at RT, permeabilized with a 0.1% saponin solution, and incubated in 50 µL of staining buffer (1% FBS containing PBS) containing a pre‐titrated optimal concentration of phycoerythrin‐conjugated anti‐human IL‐10 monoclonal antibody for 20 min at RT. The cells were washed in 2 mL of PBS and centrifuged at 300 *g* for five minutes. The pellet was resuspended in 300 µL of 2% PFA and analyzed by flow cytometry.

### 2.10 Study of IL‐6R*α* Expression on Lymphocytes and BeWo Cells

Flow cytometric analysis of IL‐6R*α* expression of lymphocytes and BeWo cells was performed by phycoerythrin conjugated anti‐human CD126 (IL‐6R*α*) antibody (BioLegend, USA). A total of 5,000 cells were analyzed per sample.

### 2.11 Detection of Soluble IL‐6R*α*

Human Instant ELISA (Thermo Fisher, USA) was used for detection of soluble IL‐6R*α* concentrations according to the manufacturer's instructions.

### 2.12 IL‐6 Pathway Signaling

The IL‐6‐induced signaling pathways were examined after IL‐6 treatment. To establish the optimal timepoint for the gene expression analysis we quantified the mRNA levels of key IL‐6 pathway factors (*IL6, IL6R, IL6ST, AKT1, STAT1, STAT3* and *PIAS3*) in lymphocytes at 2, 4, 6, 8 and 24 h after recombinant IL‐6 (Sigma‐Aldrich Co., St. Louis, USA) (0.02 ng/ mL = 5.0 × 10^7^ units/ mg) treatment. The concentration of IL‐6 was chosen accordingly to physiological circulating IL‐6 concentration in healthy pregnancy [36, 37]. Based on the results of this experiment, we chose the four‐hour treatment as the optional time point for IL‐6 pathway analysis.

For gene expression analysis, first total RNA isolated from untreated and BeWo‐12.5K lEVs‐treated lymphocytes was purified with RNeasy Mini Kit (Qiagen, CA, USA). The RNA concentration of each sample was determined using the Qubit RNA HR Assay Kit (Qubit Fluorometer, Life Technologies). Samples were stored at ‐80°C until cDNA synthesis. From each sample 600 ng total RNA was reverse transcribed with Sensifast cDNA Synthesis Kit (Bioline, London, UK). Quantitative PCR reactions using SensiFAST SYBR Hi‐ROX Kit with SYBR Green primers (Table S2) were carried out on an ABI 7900 real‐time PCR instrument (Thermo Fisher Scientific, USA) according to the manufacturer's instructions. Real‐time PCR results were calculated according to the following protocol: relative expression level = 2^−ΔCt^, where ΔCt = Ct (of gene of interest) – Ct (of houseskeeping gene) with *HPRT* as an endogenous control.

### 2.13 Flow Cytometry

Antibodies and dies used in flow cytometry are listed in Table S3. Measurements were carried out using a FACSCalibur flow cytometer (BD, San Jose, CA, USA) on the day of the staining. In the case of cellular measurements, 5 × 10^4^ cells/ tube were collected. Data were analyzed with CellQuestPro (BD, San Jose, California, USA) and Flowjo v10.3 (Tree Star Inc., OR, USA) software.

### 2.14 Statistics

The statistical analyses were performed using GraphPad Prism version 8.0 (GraphPad Software, La Jolla, CA, USA). A two‐sided unpaired Student's *t*‐test was used for normally distributed data. For multiple parameter analysis, an ANOVA test was performed, followed by a Bonferroni correction. For data that did not show normal distribution, a Mann‐Whitney U or Wilcoxon test was used. For multiple parameter analyses, an ANOVA test was performed, followed by a Kruskal–Wallis test. The level of significance was set at *p* < 0.05.

We used Statulator, a free online calculator, to estimate the sample sizes.

## 3 Results

### 3.1 Target Cells of BeWo‐EVs

The 12.5k lEVs primarily bound to CD3+ T lymphocytes; however, the binding ability of T cell subsets differed. BeWo‐lEVs preferred Th cells (5.04 ± 0.98 %) compared to Tc lymphocytes (2.01 ± 0.14 %) (*p* < 0.05). On the other hand, significantly fewer CD19+ B cells (0.92 ± 0.45 %, *p* < 0.01) and CD56+ NK cells bound BeWo‐lEVs (0.14 ± 0.05 %) (*p* < 0.001) like T lymphocytes (Figure 1A,B). There was no difference in the binding ability to the lymphocytes of pregnant and non‐pregnant donors (non‐pregnants: 3.55 ± 0.48 % vs. pregnant: 5.04 ± 0.98 %) (*p* > 0.05). We used monoclonal antibodies or AnnexinV pretreatments for blocking the BeWo‐lEVs—lymphocyte interactions (Figure 1C,D). Anti‐phosphatidylserine receptor (PSR) mAb pre‐treatment of lymphocytes partially inhibited the binding of 12.5K lEVs to CD3+/CD4+ (untreated vs. anti‐PSR Mab: 215 ± 106 mean fluorescence intensity (MFI) vs. 112.5 ± 24.06 MFI) and to CD3+/CD8+ T cells (untreated vs. anti‐PSR Mab: 33.5 ± 9.3 MFI vs. 21.43 ± 7.17 MFI). A similar effect was detected after the pretreatment of BeWo‐EVs with Annexin V (untreated vs. Annexin V treated: 13.2 ± 0.46 MFI vs. 7.06 ± 0.4 MFI). Masking of HLA‐G on BeWo‐lEVs (untreated vs. anti‐HLA‐G mAb: 13.05 ± 0.55 MFI vs. 7.83 ± 0.06 MFI) could also prevent the binding of 12.5K lEVs to lymphocytes. Neutralization of CD95 molecules on lymphocytes (untreated vs. anti‐CD95L mAb: 43.53 ±1.88 MFI vs. 39.5 ± 4.8 MFI) or CD95L (untreated vs. anti‐CD95 mAb: 13.05 ± 0.55 MFI vs. 16.35 ± 0.35 MFI) on 12.5K lEVs did not have a significant effect on the 12.5K lEV‐lymphocyte interaction. Based on these results, we concluded that interactions via PS and HLA‐G could partially coordinate the binding of 12.5k lEVs to CD4+ lymphocytes (Figure 1C,D).

**FIGURE 1:**
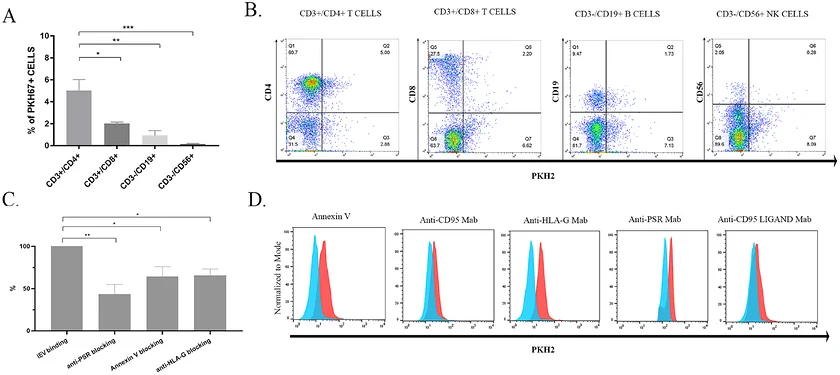
Target cells of BeWo‐derived 12.5K lEVs. (A). Binding of BeWo‐12.5K lEVs to immunophenotyped lymphocytes isolated from pregnant women BeWo‐12.5K lEVs bound significantly more CD3+/CD4+ T cells (*n* = 6 pregnant donors, one‐way ANOVA followed by Bonferroni correction). (B). Representative dot plots show the cell‐bound 12.5K lEVs to the different lymphocyte subpopulations. (C). Analysis of selected blocking between 12.5K lEVs and their target CD4+ T cells. Anti‐PSR, annexin V and anti‐HLA‐G blocking resulted in significant inhibition (*n* = 6 pregnant donors, one‐way ANOVA followed by Bonferroni correction). (D). Representative histograms show the changes of expression levels after selective blocking of a specific interaction between 12.5K lEVs and target lymphocytes (red histogram: PKH2+ surface bound 12.5K lEVs, blue histogram: PKH+ surface bound 12.5K lEVs after targeted blocking, *n* = 6 different pregnant donors; measurements were repeated 2 times from each donor).

### 3.2 IL‐6 Production Induced by BeWo‐Lymphocyte Interaction or BeWo‐Derived 12.5K lEVs

To characterize the response of lymphocytes to BeWo cells, we measured the production of the cytokines IL‐2, IFN‐γ, IL‐4, IL‐6, and IL‐17 in the cell culture supernatant. IL‐2 and IL‐17 production was detectable under baseline conditions. However, IFN‐γ, IL‐4, and IL‐6 production could not be detected in the supernatant of untreated lymphocytes.

Co‐culturing with BeWo cells induced IL‐6 production in each cell culture system that permitted either direct cell‐to‐cell interaction or cell separation by an insert. Higher levels of IL‐6 could be measured after direct cell‐to‐cell interaction than with the transwell system (direct contact: 3314 ± 191.5 pg/mL and insert separated: 2818.7 ± 126.7 pg/mL, *p* < 0.001). IL‐6 production in the co‐culture system was also validated at the mRNA level by qPCR. (Figure S6.). BeWo cells did not affect IL‐2 or IL‐17 production.

### 3.3 BeWo‐Derived 12.5K lEVs Increase the IL‐10 Production of Th Cells

Intracellular cytokine staining of CD4+ T helper (Th) cells revealed that 12.5k lEVs isolated from BeWo cells increased IL‐10 production. This change was significant compared to untreated control lymphocytes (12.5k lEV treatment: 305.9 ± 29.1 MFI; untreated CD4+ T cells: 255.3 ± 46.2 MFI; *p* < 0.01, Wilcoxon test). (Figure S7).

### 3.4 The Effects of BeWo‐Lymphocyte Interactions on the IL‐6R*α* Expression of T Cells

We investigated whether BeWo EVs affect IL‐6R*α* expression in T cells because IL‐6 induces cellular responses mediated by IL‐6R*α* expression. BeWo‐derived 12.5k lEVs significantly decreased IL‐6R*α* expression in lymphocytes (Ly+12.5k lEV: 9.7 ± 0.06 mean fluorescence intensity [MFI]) (*p* < 0.05) (Figure 2A,B). Since T lymphocytes were identified as targets of BeWo‐12.5k lEVs, we analyzed IL‐6R*α* levels in CD4+ T cell subsets. Th cells (untreated: 13.71 ± 0.68 MFI; transwell: 9.2 ± 0.04 MFI) and the CD25+ Th subset (untreated: 16.52 ± 1.18 MFI; transwell: 9.49 ± 0.27 MFI) exhibited significantly lower IL‐6R*α* expression levels when separated by insert membranes (*p* < 0.0001), as shown in Figures S8E and S8F. Direct cellular interaction (Ly + BeWo) did not have any effect.

**FIGURE 2:**
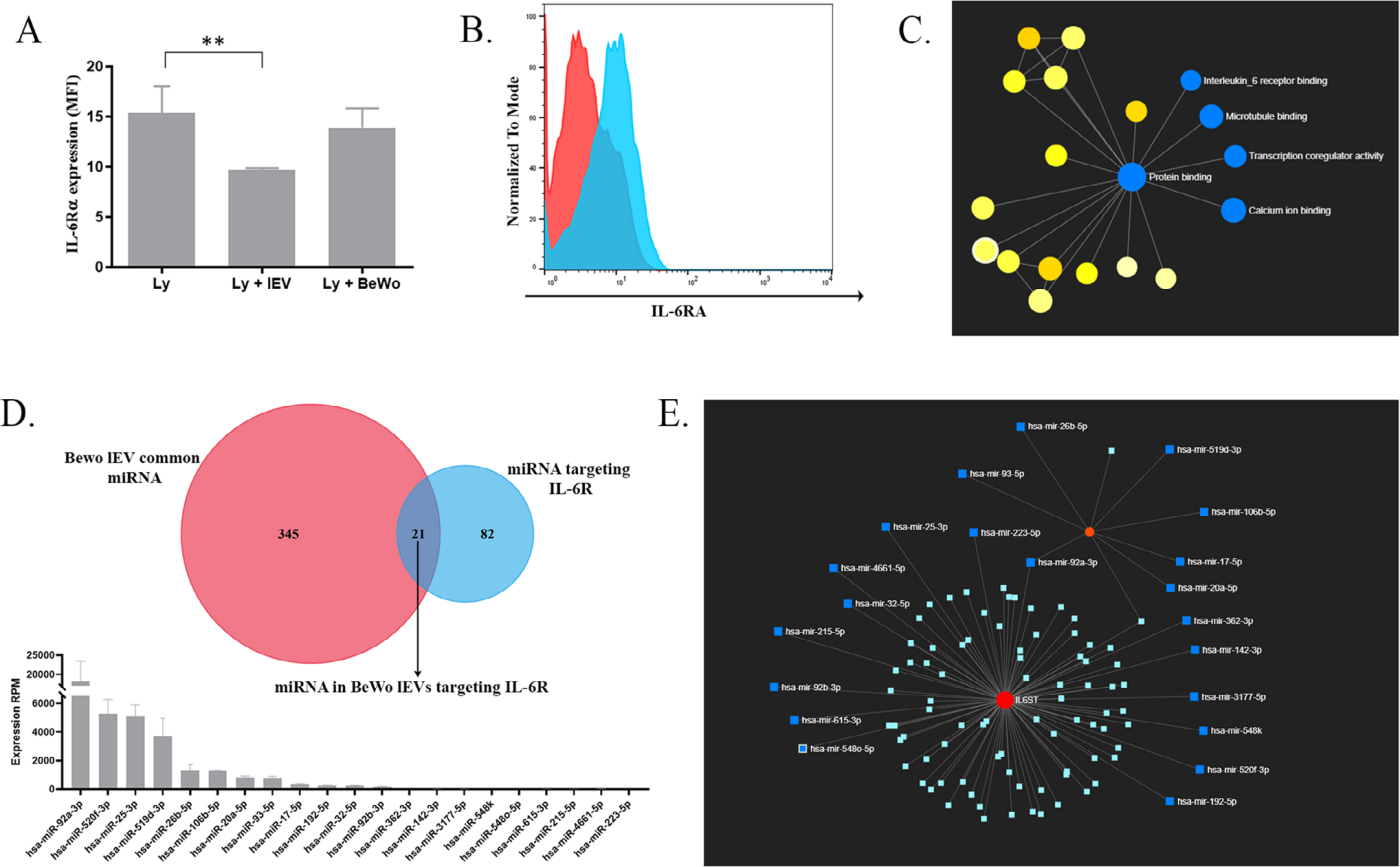
IL‐6R*α* downregulation mediated by miRNAs. (A). Analysis of IL‐6 downregulation upon BeWo‐12.5K lEV treatment (*n* = 10 donors). (B). Representative FACS histogram showing the IL‐6R*α* downregulation upon 12.5K lEV treatment marked with red (untreated control—blue) (C). Molecular function analysis of BeWo‐derived miRNAs target genes revealing the interleukin‐6 receptor binding as one of the main targets. (D). Overall, we identified 366 common miRNA in 12.5K lEVs. 21 miRNAs are targeting the downregulation of IL‐6R, together with the expression values (RPM). (E). IL‐6R downregulating miRNA network with highlighted (dark blue) miRNA found in the BeWo‐12.5K lEVs. Values represent mean ± SEM (*n* = 10 ** *p* < 0.01, *** *p* < 0.001) Ly—lymphocytes.

Decreased IL‐6R*α* expression can be caused by receptor shedding or decreased gene expression. Soluble IL‐6R*α* can be formed by ectodomain shedding or alternative splicing. However, cells can also lose their exofacial IL‐6R*α* through EV release [38]. To determine the mechanism of decreased IL‐6R*α* expression, the concentration of soluble IL‐6R*α* and also the EV‐bound IL‐6R*α* was determined by ELISA or flow cytometry, respectively. Treatment of lymphocytes with BeWo‐derived 12.5K lEVs did not change either the soluble IL‐6R*α* concentration in cell culture supernatant (untreated vs. 12.5K lEV treated: 114 ± 45.8 pg/ mL vs. 178.3 ± 24.1 pg/ mL *p* > 0.05) or the number of IL‐6R*α* positive 12.5K lEVs (untreated vs. 12.5K lEV treated: 741 ± 326 events/ µL vs 1071 ± 351 events/ µL, data not shown), which may suggest that the IL‐6R*α* downregulation could happen at the gene expression level.

### 3.5 IL‐6R*α* Signaling in Lymphocytes

The IL‐6Rα signaling pathway was identified in lymphocytes following treatment with recombinant IL‐6. *STAT1* showed a 3.5‐fold change increase in mRNA expression upon 12.5K lEVs treatment (relative gene expression of untreated: 265.8 ± 125.8, 12.5K lEVs treated: 1014 ± 102.4, Ly + BeWo: 308.4 ± 148.3, Ly insert BeWo: 990.8 ± 75.2). *PIAS3* showed a 10 fold decrease in 12.5K lEVs treated lymphocytes (relative gene expression of untreated: 44.7 ± 0.07, 12.5K lEVs treated: 4.48 ± 0.017, Ly + BeWo: 1.09 ± 0.37, Ly insert Bewo: not detected), *AKT1* showed an 1.4 fold increase upon 12.5K lEVs stimulation (relative gene expression of untreated: 16.8 ± 0.68, 12.5K lEV treated: 23.3 ± 0.4, Ly + BeWo: 17.39 ± 3.15, Ly insert BeWo: 21.15 ± 4.0), *RELA*, *NFKBIA* showed an increased mRNA expression (Figure 3). In BeWo‐lymphocyte co‐culture system we detected a decrease in *CXCL12*, *IL1R1*, *IL6ST*, *OSMR*, *TNFRSF10B* and *STAT3* expression. Separation of BeWo cells and lymphocytes with 1 µm pore sized membrane led to increased *IL10*, *IL2RA*, *IL1R1*, *CD80*, *JAK2*, *JAK3*, *MAPK1*, *MAPK14*, *RAC1* and decreased *STAT3* expression detectable in lymphocytes (Table S4).

**FIGURE 3:**
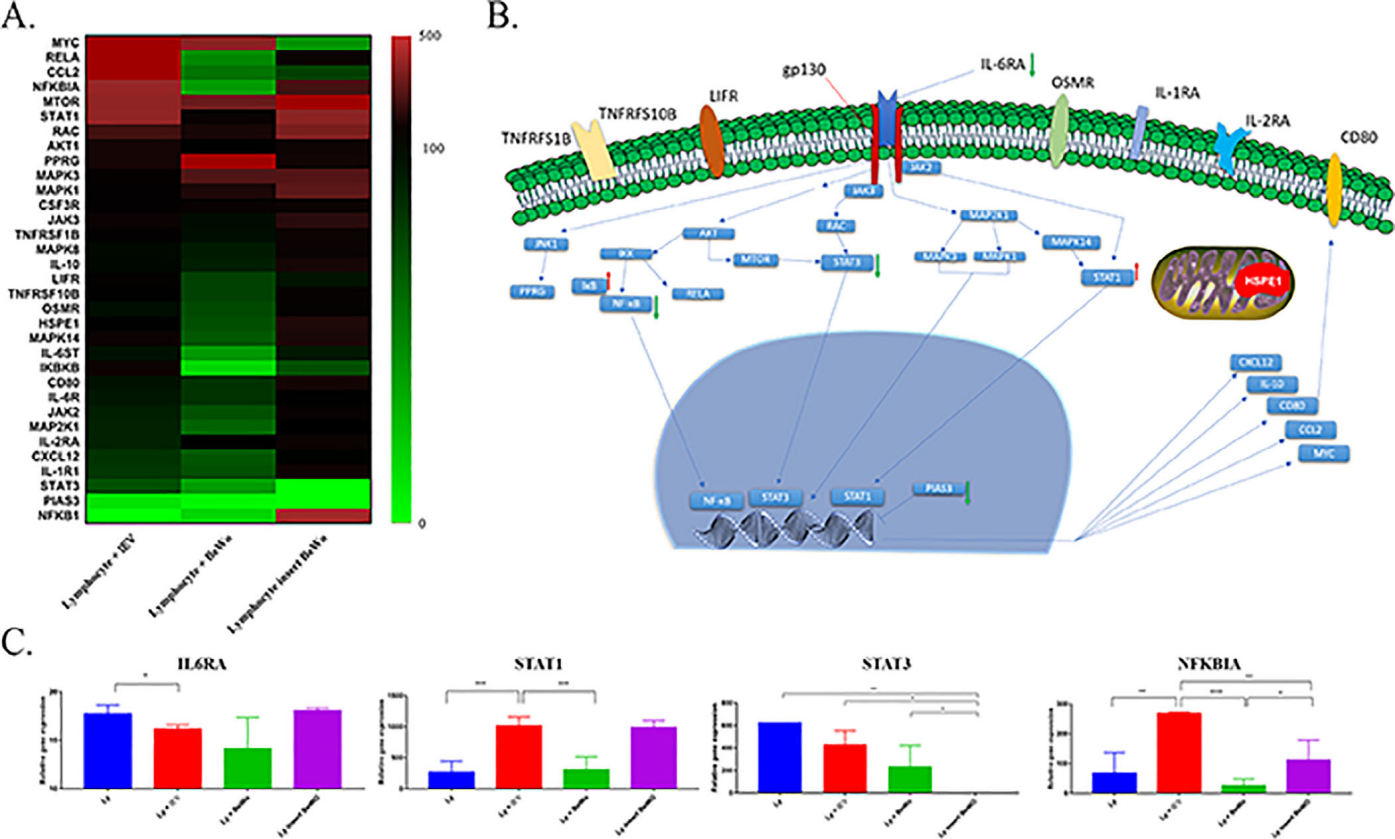
IL‐6 pathway analysis. (A). Heatmap analysis of IL‐6 pathway of non‐pregnants’ lymphocytes treated with BeWo‐12.5K lEVs, lymphocytes co‐cultured with BeWo cells with or without a 1 µm pore sized insert membrane (*n* = 3, each sample was run in triplicates). Heatmap showing the differential expressed genes of 12.5K lEV treated or BeWo co‐cultured lymphocytes normalized to the untreated control lymphocytes. (B). Schematic figure of the analyzed IL‐6 pathway molecules. Green arrows show significant downregulation at mRNA level upon BeWo‐derived 12.5K lEV treatment. Red arrows mark the upregulated molecules upon 12.5K lEV treatment. (C). Figures showing the relative gene expression: down‐ or upregulation of *IL6R*, *STAT1, STAT3* and *NFKBIA* mRNAs upon different treatments of lymphocytes (one‐way ANOVA followed by Bonferroni correction, * *p* < 0.05, ** *p* < 0.01, *** *p* < 0.001, ****p < 0.0001).

### 3.6 MiRNA Content of BeWo‐Derived LEVs

To investigate the mechanism of IL‐6 downregulation we performed miRNA‐Seq of BeWo‐derived 12.5K lEVs. Altogether we identified 517 ± 70 miRNAs in the 12.5K lEV samples, out of which 366 were common in all samples. The biological pathway analysis revealed that the interleukin‐6 receptor binding (GO:0005138) to be significantly enriched (*p*_adj_ = 0.02) (Figure 2C) and 21 miRNAs from the 366 were targeting IL‐6R. Overall, in the NetworkAnalyst database there are 103 known miRNAs targeting IL‐6R, through gene silencing of *IL6ST* and *ERAP*. The top 5 highly expressed miRNAs targeting IL‐6R found in BeWo‐derived 12.5K lEVs were the hsa‐miR‐92a‐3p (18170 ± 5221 RPM), hsa‐miR‐520f‐3p (5274 ± 1002 RPM), hsa‐miR‐25‐3p (5065 ± 838.8 RPM), hsa‐miR‐519d‐3p (3703 ±1261 RPM) and hsa‐miR‐26b‐5p (1348 ± 363.3 RPM) (Figure 2D,E).

Further analysis of the miRNA pattern revealed that there is a strong miRNA signature pattern among the biological replicates (Figure 4A,C). Most of the target genes of the miRNAs found in BeWo‐12.5K lEVs are expressed in the placental tissue and one of the main target cell types is the CD4+ T cell (Figure 4B). Beside the IL‐6 signaling pathway, other significantly enriched targeted biological pathways were FAS and Arf6 downstream pathways, the plasma membrane estrogen receptor and VEGF signaling, followed by the β1integrin cell surface interaction biological pathway (Figure 4D). Additionally, BeWo‐12.5K lEVs induced molecular function network analysis reveals cell differentiation as one of the main significant molecular functions (*p* = 0.001, Figure 4E). The in‐silico analysis of cell differentiation depicts that the process is initiated by signal transduction (IL‐6R downregulation) followed by regulation of cell proliferation and cell cycle coupled with anti‐apoptotic signaling events. Furthermore, cell growth and immune response balancing accompany cell differentiation (Figure 4F).

**FIGURE 4:**
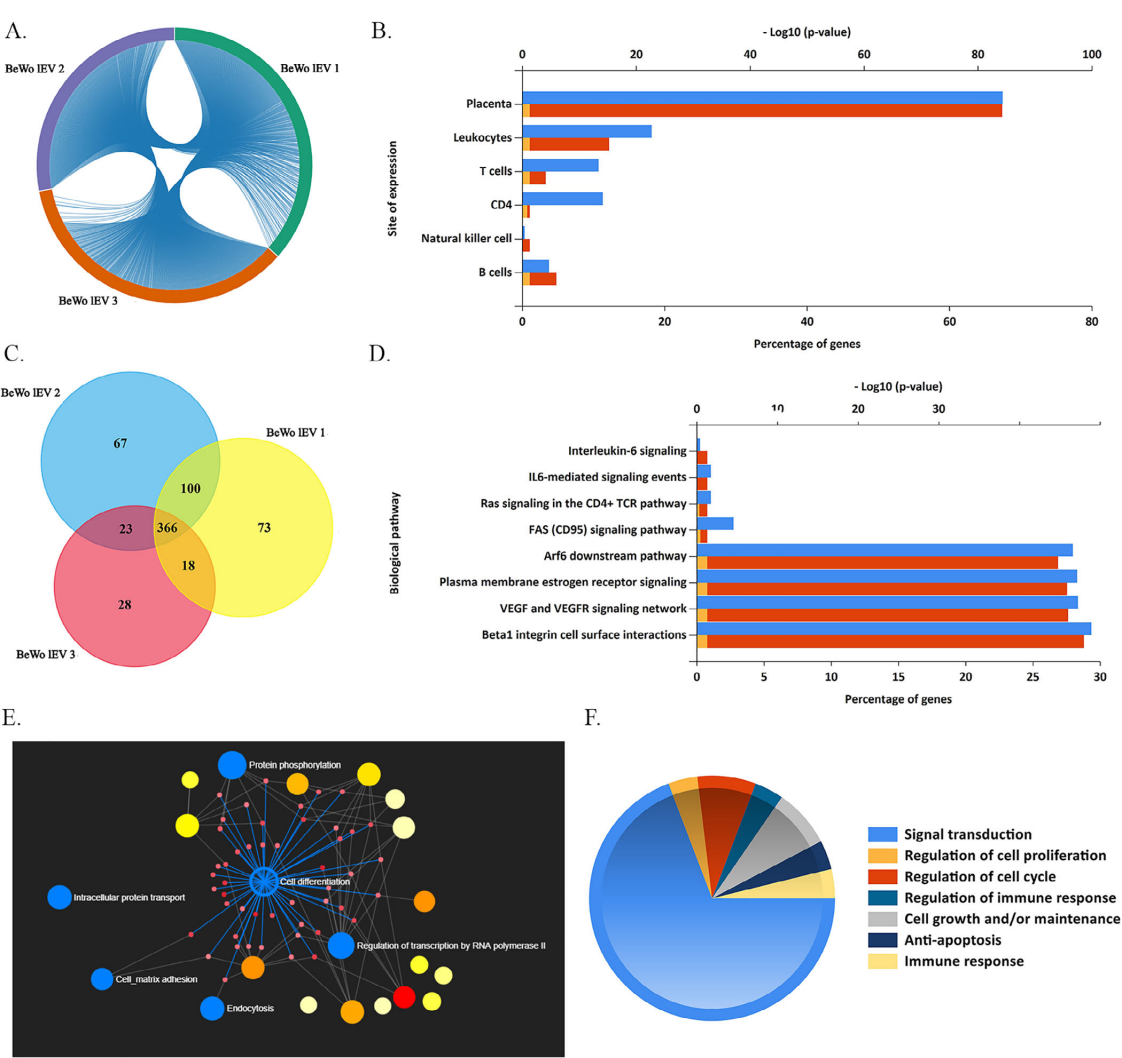
BeWo‐derived 12.5K lEV miRNA content. (A). Chord diagram analysis showing the overlap between the biological replicates. (B). Bar chart showing the site expression of genes targeted by BeWo‐12.5K lEVs. Based on the analysis the main target tissue is the placenta. One of the prominent cell types are the leukocytes, most of the targeted cells are the CD4+ T cells (blue bars represent the percentage of target genes, red bars are representing the p‐values, and the yellow bars represent the *p* = 0.05 reference). (C). Venn diagram showing the 366‐common miRNA in 12.5K lEVs. (D). Biological pathway analysis showing the miRNA targeted pathways, among the IL‐6 signaling, the following pathways are significantly targeted: FAS signaling and Arf6 downstream pathways, the plasma membrane estrogen receptor and VEGF signaling. E. BeWo‐derived 12.5K lEV induced molecular function network analysis reveals as the main molecular function the cell differentiation. (F). The in‐silico detailed analysis of cell differentiation function reveals the molecular function is mainly induced through alteration of signal transduction (IL‐6R downregulation), followed by regulation of cell proliferation and cell cycle coupled with an anti‐apoptotic signaling event. Furthermore, cell growth and immune response balancing accompany the cell differentiation.

### 3.7 IL‐6R*α* Expression of Circulating Lymphocytes of Pregnant and Non‐Pregnant Women

As our in vitro studies showed a strong IL‐6R*α* downregulation in CD4+ lymphocytes, we aimed to assess the in vivo significance of IL‐6R*α* downregulation during pregnancy. Peripheral blood samples from first trimester healthy pregnant and non‐pregnant control women were assessed for IL‐6R*α* expression on circulating lymphocytes. IL‐6R*α* expression was detected in all tested T cell subsets. However, the proportion of IL‐6R*α*+ cells varied among them. Since IL‐6 sensitivity depends on the number of IL‐6R*α* receptors, we compared their expression levels in T cell subsets. Exofacial IL‐6R*α* was significantly higher on CD4+ T cells (13.72 ± 0.68 MFI) than on CD8+ T cells (9.31 ± 0.16 MFI). (*p* < 0.0001) (Figure S8A‐D).

We detected the significantly lower percentage of IL‐6R*α* expressing CD3+/CD4+/CD25+ lymphocytes in the blood of pregnant women compared to the healthy non‐pregnant group (81.53 ± 4.2 % vs. 92.78 ± 1.95 %; *p* < 0.05) (Figure 5A–D).

**FIGURE 5:**
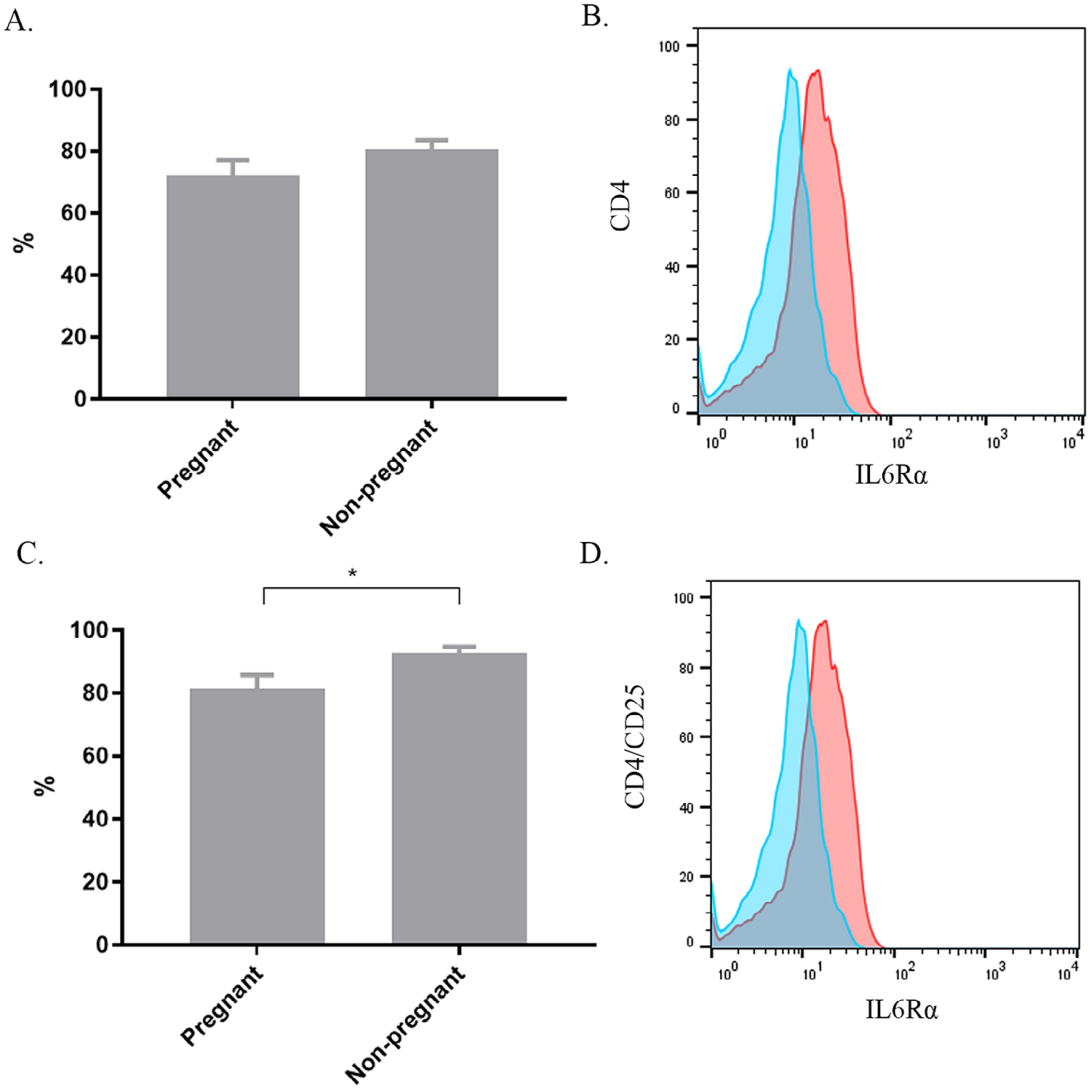
IL‐6R*α* expression on pregnant and non‐pregnant lymphocytes. (A). Analysis of IL‐6R*α* expressing circulating CD4+ T cells in healthy non‐pregnant and pregnant donors (*n* = 12). (B). Representative overlay histograms show the IL‐6R*α* expression on CD4+ lymphocytes of a pregnant (blue histogram) and a non‐pregnant donor (red histogram). (C). Percentage of IL‐6R*α* expressing CD4+/CD25+ T_reg_ cells in healthy non‐pregnant and pregnant donors (*n* = 12). D. Representative overlay histograms show the IL‐6R*α* expression on CD4+/CD25+ lymphocytes of a pregnant (blue histogram) and a non‐pregnant donor (red histogram) (unpaired *t*‐test, * *p* < 0.05).

## 4 Discussion

A successful pregnancy depends on the immunological recognition of the semi‐allogeneic fetus and the subsequent development of maternal immunotolerance. This process involves strictly regulated feto‐maternal interactions, including cellular and humoral factors, as well as EVs. One of the key players in maintaining a tolerant immune environment is the regulatory T cell (Treg). Tregs are characterized by their CD3+/CD4+/CD25high+/FoxP3+/CD127‐ phenotype and their production of TGF‐*β* and IL‐10 [39, 40].

Treg cells are present at the feto‐maternal interface and in the bloodstream during pregnancy. However, their number, phenotype, and suppressive activity undergo dynamic changes [41, 42, 43]. Elevated levels of Treg cells can be detected in local lymph nodes and vaginal secretions even in the earliest weeks of gestation. This raises the question of whether the early presence of regulatory T cells in pregnant tissues is a consequence of local differentiation from naïve T cells. One theory suggests that fetal antigens, presented by local professional antigen‐presenting cells, induce the differentiation of CD4+/CD25‐ T helper (Th) lymphocytes into CD4+/CD25+ regulatory T (Treg) cells [44, 45].

At the feto‐maternal interface, trophoblast cells present fetal alloantigens. Through the expression of non‐classical MHC‐I, trophoblasts inhibit the activity of effector T cells and NK cells, thereby promoting a tolerogenic immune environment. Among the immunoregulatory molecules associated with EVs derived from placental extravillous trophoblasts, one of the most important is HLA‐G, a non‐classical MHC‐I molecule. HLA‐G plays a key role in establishing maternal immune tolerance by inhibiting NK cells via ILT2 and ILT4 receptors, inhibiting antigen presentation and promoting the differentiation of local Treg cells [46, 47, 48, 49].

The role of HLA‐G in inducing Treg cells is well known. It is also widely recognized that EVs derived from trophoblasts expressing HLA‐G mediate this effect [50].

In previous studies, we demonstrated that 12.5k lEVs derived from trophoblasts, expressing HLA‐G and isolated from maternal plasma, bind to T lymphocytes. This binding inhibits the IL‐2‐induced signaling pathway in T cells [28].

In another experiment, we developed a BeWo cell line with a CRISPR‐Cas9‐based HSPE1 knockout and demonstrated that trophoblast‐induced Treg differentiation depends on EV‐associated HSPE1 [29]. Based on these findings, we investigated the effects of BeWo cells and BeWo‐derived EVs on cytokine production in relation to regulatory T cell differentiation.

We established in vitro model systems in which BeWo cells and lymphocytes were cultured together. We also used a Transwell system that separated the BeWo cells with a 1‐µm insert to prevent direct cell‐to‐cell interaction while still allowing communication via EVs. Co‐culturing of BeWo cells and lymphocytes resulted in IL‐6 production. IL‐6 shifts T‐cell polarization towards the Th17 phenotype [16], which is detrimental to pregnancy. This contradicted the results of our previous experiments, in which we demonstrated that trophoblast‐derived EVs promote Treg‐like polarization through their HSPE1 content [29]. To resolve this apparent contradiction, we examined the sensitivity of T cells to IL‐6 by analyzing IL‐6R *α* chain expression.

In our experiments, BeWo‐derived EVs were found to decrease IL‐6R*α* expression on CD4+ T cells. The expression of cell surface receptors can be regulated through various pathways, including ectodomain cleavage [51], vesicle‐mediated removal [52], and transcriptional or posttranscriptional modifications [53]. Based on our experiments, we ruled out ectodomain cleavage and vesicle‐mediated receptor removal. Instead, we focused on transcriptional regulation. We hypothesized that this regulation occurs through alterations in the IL‐6 pathway, resulting in decreased IL6R mRNA levels. We confirmed this hypothesis via quantitative polymerase chain reaction (qPCR) analysis. We also examined the IL‐6 signaling pathway after treating with recombinant IL‐6. Our goal was to determine if the BeWo‐EV‐induced alteration in IL‐6R*α* could be attributed to a change in this pathway. Increased expression of IL‐10, IL‐2RA, IL‐1R1, CD80, JAK2, JAK3, MAPK1, MAPK14, and RAC1, along with decreased STAT3 expression, was observed in lymphocytes. This suggests reduced IL‐6 signaling and Treg‐like polarization.

Due to their molecular composition, EVs act as complex intercellular signaling molecules. The function of target cells is influenced by both their surface‐associated and intravesicular molecules.

To elucidate the mechanism by which IL6R is downregulated, we analyzed whether miRNAs are mediating the observed process. miRNAs are a class of small, non‐coding RNA molecules that play a major role in post‐transcriptional regulation of protein expression [53]. Using next‐generation sequencing, we analyzed the miRNA profiles of BeWo‐12.5K lEVs and identified 21 miRNAs that target the downregulation of IL6R through gene silencing. We performed target enrichment analysis to predict the function of miRNAs. First, we retrieved all predicted targets for the significantly changed miRNAs detected and submitted the target list for Gene Ontology (GO) enrichment analysis. We detected 366 microRNAs (miRNAs) in the BeWo‐EV samples. We categorized these using the NetworkAnalyst database. The database lists 103 miRNAs associated with IL‐6R signaling. After comparing them with the BeWo‐EV miRNA profile, we found that the five most highly expressed miRNAs in this group were hsa‐mir‐92a‐3p (18,170 ± 5,221 RPM), hsa‐mir‐520f‐3p (5,274 ± 1,002 RPM), hsa‐mir‐25‐3p (5,065 ± 838.8 RPM), hsa‐mir‐519d‐3p (3,703 ± 1,261 RPM), and hsa‐mir‐26b‐5p (1,348 ± 363.3 RPM).

Our findings suggest that HLA‐G‐expressing trophoblastic 12.5k lEVs play a central role in regulating local T helper (Th) cell polarization by modulating their sensitivity to interleukin (IL)‐6. The presence of placental EVs in the maternal circulation suggests that they may also play a role in maintaining maternal immune tolerance at the systemic level. The lower circulating level of the IL‐6 receptor alpha (IL‐6R*α*) bearing CD4+/CD25+ T cell subset in healthy pregnant women supports this hypothesis.

In the placental microenvironment, EV‐mediated interactions between trophoblast cells and lymphocytes orchestrate the local immune response, which is critical for maintaining maternal immune tolerance during pregnancy.

## Conflicts of Interest

The authors declare no conflicts of interest.

## Supporting information




**Supporting Information**: aji70335‐sup‐0001‐SuppMat.docx

## Data Availability

The data that support the findings of this study are available from the corresponding author upon reasonable request.
